# Concurrent validity of lower extremity kinematics and jump characteristics captured in pre-school children by a markerless 3D motion capture system

**DOI:** 10.1186/s12998-019-0261-z

**Published:** 2019-08-11

**Authors:** Steen Harsted, Anders Holsgaard-Larsen, Lise Hestbæk, Eleanor Boyle, Henrik Hein Lauridsen

**Affiliations:** 1 Research Unit for Clinical Biomechanics, Department of Sports Science and Clinical Biomechanics, University of Southern Denmark, Campusvej 55, 5230 Odense M, Denmark; 2 Orthopaedic research unit, Department of Orthopaedic Surgery and Traumatology, Odense University Hospital, Odense, Denmark; 3 Department of Clinical Research, University of Southern Denmark, Odense, Denmark; 4 Nordic Institute of Chiropractic and Clinical Biomechanics, University of Southern Denmark, Campusvej 55, 5230 Odense M, Denmark

**Keywords:** Markerless motion capture, Concurrent validity

## Abstract

**Background:**

Investigations into the possible associations between early in life motor function and later in life musculoskeletal health, will require easily obtainable, valid, and reliable measures of gross motor function and kinematics. Marker-based motion capture systems provide reasonably valid and reliable measures, but recordings are restricted to expensive lab environments. Markerless motion capture systems can provide measures of gross motor function and kinematics outside of lab environments and with minimal interference to the subjects being investigated. It is, however, unknown if these measures are sufficiently valid and reliable in young children to warrant further use. This study aims to document the concurrent validity of a markerless motion capture system: “The Captury.”

**Method:**

Measures of gross motor function and lower extremity kinematics from 14 preschool children (age between three and 6 years) performing a series of squats and standing broad jumps were recorded by a marker-based (Vicon) and a markerless (The Captury) motion capture system simultaneously, in December 2015. Measurement differences between the two systems were examined for the following variables: jump length, jump height, hip flexion, knee flexion, ankle dorsi flexion, knee varus, knee to hip separation distance ratio (KHR), ankle to hip separation distance ratio (AHR), frontal plane projection angle, frontal plane knee angle (FPKA), and frontal plane knee deviation (FPKD). Measurement differences between the systems were expressed in terms of root mean square errors, mean differences, limits of agreement (LOA), and intraclass correlations of absolute agreement (ICC (2,1) A) and consistency of agreement.

**Results:**

Measurement differences between the two systems varied depending on the variables. Agreement and reliability ranged from acceptable for e.g. jump height [LOA: − 3.8 cm to 2.2 cm; ICC (2,1) A: 0.91] to unacceptable for knee varus [LOA: − 33° to 19°; ICC (2,1) A: 0.29].

**Conclusions:**

The measurements by the markerless motion capture system “The Captury” cannot be considered interchangeable with the Vicon measures, but our results suggest that this system can produce estimates of jump length, jump height, KHR, AHR, knee flexion, FPKA, and FPKD, with acceptable levels of agreement and reliability. These variables are promising for use in future research but require further investigation of their clinimetric properties.

## Background

The easy, valid, and reliable capture of gross motor function and lower extremity kinematics in young children may have a wide range of applications within both research and clinical practice. Such applications may include investigations into the possible short and long-term associations between motor function and musculoskeletal health. At present optoelectronic marker-based systems provide reasonably valid [1–4] and reliable [5, 6] measurements of human movement, but does so at the price of a costly lab setup, long participant preparation times, and the unfeasibility of attaching markers in certain settings [7–9]. Markerless motion capture has now technically matured to the point where it provides a potentially promising solution to the investigation of human movement, often by the use of cutting-edge developments within computer vision and machine learning [7]. Markerless motion capture allows for the easier capture of human movement, both within and outside of a laboratory setting, and does so with minimal interference to the movements being investigated [7]. The validity of some three-dimensional (3D) markerless systems have been examined in adult populations [10–12], but to our knowledge, no markerless 3D motion capture system has been validated for use in young children.

**Fig. 1:**
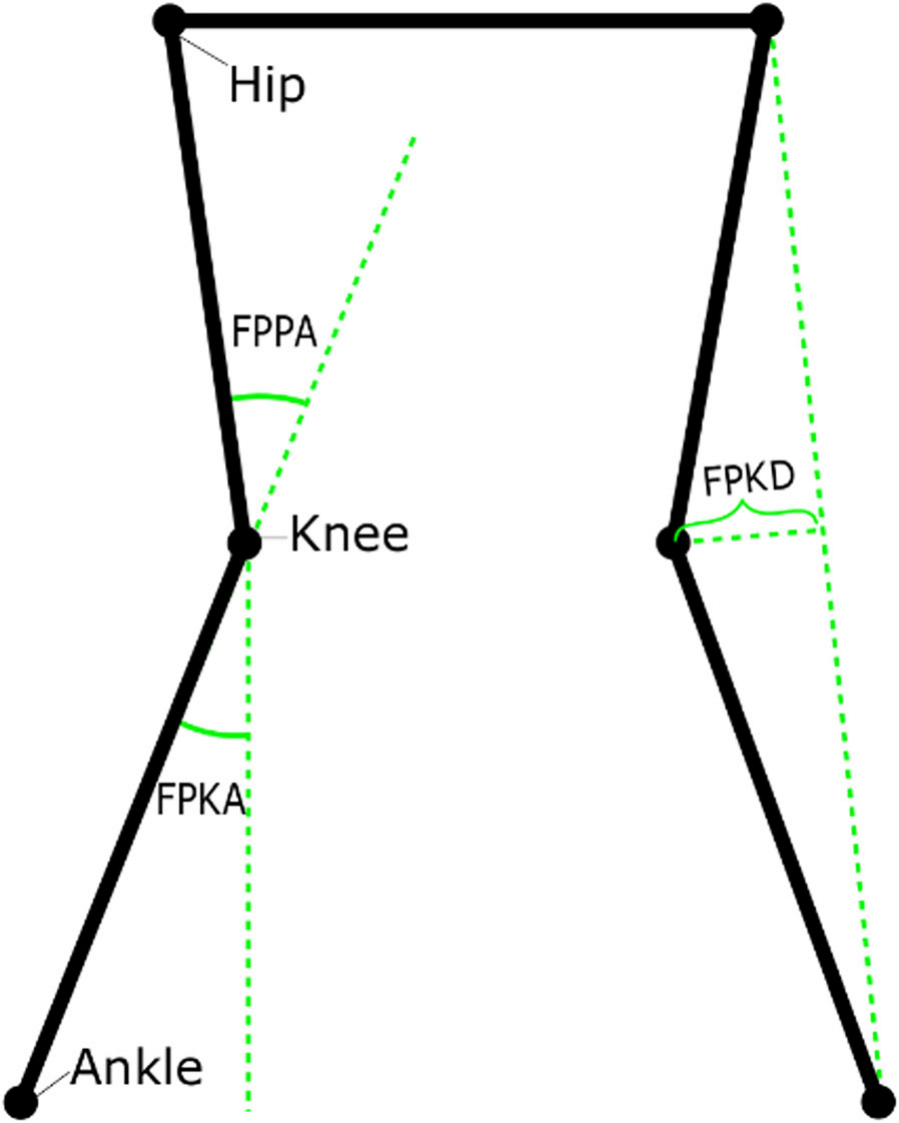
Frontal Plane Measures. Note: Hip, knee, and ankle joint center positions projected onto the frontal plane. Frontal Plane Projection Angle (FPPA), Frontal Plane Knee Angle (FPKA) and Frontal Plane Knee Deviation (FPKD) are calculated based on these projected joint center positions. The shown positions of the joint centers represent the medial deviation of both knees during a squat

The potential associations between motor function and musculoskeletal health has typically been explored by: marker-based measures of knee, hip and ankle dorsiflexion (sagittal plane) [13], and knee varus/valgus (frontal plane) [14–16]; two-dimensional (2D) planar measures suitable for single camera approaches (Fig. 1); or manual measures of jump length [17, 18]. The recent developments in modern markerless systems will now allow for the easy capture of all these measures, but to date, these measures have not been validated in young children.

Therefore, our objective was to investigate and evaluate the concurrent validity of kinematic variables and performance measures related to lower extremity gross motor function evaluated by a 3D markerless motion capture system (the Captury system) as compared to a marker-based system (Vicon system) in a sample of preschool children.

## Methods

### Study population

The study population consisted of a convenience sample of 14 preschool children who attended a preschool near the test facilities at The University of Southern Denmark. Inclusion criteria were consenting children aged from 3 to 6 years with no known illness or disease. Before inclusion, written information was given to both the preschool and parents (by LH and HHL), and written informed consent was collected and verified from the parents by LH and HHL. For descriptive purposes, age and sex of the study population were recorded. The study follows the ethical laws of Denmark. The data was collected in December 2015.

### The Vicon and Captury systems

*The Vicon system* (Vicon Motion Systems INC, Oxford, UK) [19] is a widely used marker-based 3D motion capture system. Marker-based systems, including the Vicon, can capture kinematics in children at the age of 5 to 15 years with acceptable test-retest reliability [5, 6, 20, 21]. Our setup consisted of eight MX-T20 (2 megapixels), eight MX-T40 (4 megapixels) and two Bonita digital cameras (1 megapixel). The operating software was Nexus (version 2.3) [22]. The sampling rate was 200 Hz for the 16 infrared and 50 Hz for the two digital cameras. On both test days, a full calibration including all cameras in the Vicon system was conducted using an active wand. Wand count collection was stopped at 3000 and 500 wand counts for the MX and Bonita cameras, respectively. The image error was below 0.2 mm and 0.45 mm for the MX and Bonita cameras, respectively on both test days. All gap-filling was done manually using “Rigid Body” and “Pattern Fill”. Trajectory data was filtered using a Woltring Filter (mean square error of 10 mm^2^). System calibration and data processing were done by a biomedical engineer with training and experience in using the Vicon system.

*The Captury system* (The Captury GmbH, Saarbrüken, Germany) [23] is a fast set-up, markerless, and optical 3D motion capture system based on traditional commercial video cameras. The Captury is based on a passive vision system [8] that uses a visual hull [24] and a background subtraction method [25] to estimate the silhouette of the subject being captured. A Captury-specific template skeleton is fitted into this set of silhouettes, and the template skeleton is then transformed via an automatic scaling process into a subject-specific skeleton. This automatic process involves estimating joint center positions by use of multiple 3D Gauss functions and local optimization procedures [26] and is usually completed within 1 min. Our setup consisted of eight Go-Pro cameras mounted on tripods in an oval (5 m × 6 m) around the recording area. The sampling rate was 50 Hz. Calibrations of the Captury system was done at the beginning of both test days using the standard calibration board [27]. All cameras had between 50 and 120 board detections. The image error was 1.3 mm and 2.7 mm for day one and day two, respectively. All calibrations and recordings related to the Captury were handled by the Executive Director and developer of the system (Dr. Nils Hasler).

The recorded files were processed using the software CapturyLive [23] version 1.0.135. The recordings were retracked using the setting “very high” [27], and data was exported using standard export options meaning that no filter was applied to the data. The average illuminance of the recording area was 246 lx (mean of 8 measures; standard deviation 30 lx).

Neither of the two concurrently recording systems is believed to have affected the other system.

### Test procedure

Upon arrival, an instructor (SH) gave the children a common introduction to the test-setup and the process of positioning the reflective markers.

#### The positioning of the reflective markers

Anthropometric measurements needed for the Vicon system were taken, and 23 14 mm Vicon reflective markers were placed in accordance with the Plug-in Gait marker placement procedure [28] on their feet, ankles, legs, pelvis, torso, and shoulders. To improve rotational measures, wands were used for the femoral and tibial markers. Immediately prior to recording, and by use of a cross-line laser, it was assured that the femoral wand marker was positioned in the plane of the hip joint center and knee joint center and that the tibial wand marker was positioned in the plane of knee joint center and ankle joint center. The process of placing the reflective markers was performed by a team of two experienced users of the Vicon system with the help of two experienced clinicians. At least one experienced user was involved in the marker placement for each child.

#### Recordings

Each child completed a series of five functional tests in the following order; squats, vertical jumps, box drops, drop vertical jumps and standing broad jumps. These tests were chosen as they are simple, functional, and can capture physical performance. Furthermore, valid and reliable measures of the mechanics involved in landing may have value in future investigations into the potential associations between movement patterns and musculoskeletal health [16, 29]. Each test was repeated three times consecutively. This study exclusively reports on the squats and standing broad jumps, as it was assumed that these tests represent the extremes in terms of changes in spatial position and speed.

##### Squat procedure

The examiner (HHL) was standing outside the center of the capture volume of the two systems and facing the child in the center. The child was instructed to do as the examiner who performed the squat. For the squat, the feet were placed shoulder width apart with arms stretched out in front of the body and parallel to the floor and a deep squat was performed.

##### Standing broad jump procedure

The examiner was standing outside the long end of the capturing volume and faced the child who was positioned approximately 1 meter behind the center of the volume. The child was then instructed to jump simultaneously with both legs as far forward as possible. No instructions on arm movements were given.

#### Event marking and synchronization of recordings

To synchronize the two systems to identical start and end points a flash from an LED light-signal was given before and after each repetition of the movements. Subsequently, the Vicon data was downsampled from 200 Hz to 50 Hz to match the sampling frequency of The Captury system.

For several of the movements, there was a considerable period from the flash of the LED light-signal until the movement was initiated by the subject. In order to remove this period from the recordings, squats were trimmed using an acceleration-based algorithm, and the standing broad jumps were trimmed to include from the deepest part of the preparation phase to the deepest part of the landing phase.

For the jumps, events related to ground contact were marked using visual analysis of the video-recordings as force plates were not available in the Captury system. Since the Captury system provided visual information from eight directions, whereas the Vicon only provided optical information from two directions, it was decided that marked time points obtained from the visual analysis of Captury data would be used for the Vicon data as well.

For all jumps, two frames were marked: (1) Toe-Off, the last frame where one or more toes still had contact with the floor; (2) Full-foot-contact, the first frame where one of the feet was placed flat on the floor.

### Measured variables

In addition to sagittal plane kinematics (hip flexion, knee flexion, and ankle-dorsi flexion) and frontal plane knee varus, several planar measures calculated from joint-center positions projected onto the frontal plane were compared. The frontal plane for these projected measures was defined as the plane between the two hip-joint centers perpendicular to the ground plane. The ground plane was derived by using the length and width coordinates (no height coordinates) from both systems.

*Frontal Plane Knee Angle (FPKA)* FPKA captures the angle in the frontal plane between a unit vector going from the center of the knee joint to the center of the ankle joint, and a unit vector going from the knee joint straight down [30, 31] (Fig. 1). FPKA has been proposed as a potential screening tool for the assessment of frontal plane knee kinematics due to its correlation with knee varus and high reliability [30, 31].

*Frontal Plane Projection Angle (FPPA)* captures the angle in the frontal plane between a unit vector going from the hip joint to the knee joint, and a unit vector going from the knee joint to the ankle joint [32] (Fig. 1). The FPPA has been used to document increased risk of acute lower extremity injury [33], and has been proposed as a potential cost-effective screening alternative to 3D analysis for the assessment of frontal plane knee kinematics, as the measure was found to be both highly correlated with 3-D measures of knee valgus and reliable [30, 34].

*Frontal Plane Knee Deviation (FPKD)* is measured in the frontal plane as the shortest possible distance from the knee-joint center to a line between the ankle-joint and hip-joint centers, with negative values indicating the knee being placed medially to the hip-ankle line and positive values indicating the knee being placed laterally to the line (Fig. 1). FPKD has been used to express medial knee displacement during the landing phase of drop vertical jumps [15].

*Knee-Hip Separation Distance Ratio (KHR)* is calculated as the distance between the knee joint centers divided by the distance between the hip joint centers. *Ankle-Hip Separation Distance Ratio (AHR)* is calculated as the distance between the ankle joint centers divided by the distance between the hip joint centers [35]. KHR and AHR have been used to assess the effect of neuromuscular training interventions targeted at changing frontal plane knee kinematics in adolescents [30, 36].

*Jump length* was calculated, in the ground plane, as the distance from a point directly between the two ankle-joints at the frame marked with “toe-off” to the center of the ankle joint with the lowest position at the frame marked with first flat foot contact. Manual measures of jump length have been shown to be reliable over both short- and long-term [17, 18], and to correlate well with other measures of physical performance [17].

*Jump height* was calculated as the difference between the average height of the hip-joints at toe off and the highest average position of the hip-joints during the phase of the jump between toe-off and full-foot-contact.

### Measurement types

For all variables except jump length and jump height, we report on three different measurements: ‘peak’, ‘point’, and ‘through range’. The peak measurements refer to the maximum and the minimum values of a given variable throughout the entire movement. The point measurements refer to measures obtained at a specific point during the movements such as the deepest position of a squat or the moment of landing during jumping. Finally, the ´through range´ specifies all points measured throughout the full motion. All measurements were calculated independently for each system. For the standing broad jumps the points selected for analysis was the moment of landing, defined as being the frame marked with “full-foot contact”, and the deepest position during the landing phase. For the squats, the following two points were selected for analysis: 1) The deepest position of the squat, defined as the frame with the highest value of knee flexion, and 2) The mid-range position of the squat, defined as the frame during descent were knee-flexion was closest to half of its peak value during the same repetition; i.e., it is a comparison of the values measured by the two systems when the child is halfway down during the squat.

For the unilateral measures, only values from the left leg are presented in the present study, since the differences between the left and right leg were negligible.

### Statistical analyses

The study uses the definitions of reliability and agreement suggested by GRASS (Guidelines for Reporting Reliability and Agreement Studies) [37]. Agreement will be discussed using the terms accuracy and precision as defined by Rodrigues [38].

Age, height, and weight of the 14 preschool children were described using means and standard deviations (SD).

For all variables and measurement types (peak, point, and through range), agreement and reliability between the Vicon and Captury systems were visualized by Bland and Altman plots, and 2-dimensional scatter plots supplied with a line of equality. The assumption of homoscedasticity was tested via assessment of Bland-Altman plots. When heteroscedastic relationships were found, a natural log transformation was considered before further statistical analysis.

Estimates of reliability and agreement were made by analyzing concurrent measurements of peak values, point values, and through range motion for the different angles obtained from the squats and standing broad jump tests. Different statistical approaches are required for point and peak values and through range motion.

### Peak and point value analysis

For both peak and point values, limits of agreement (LOA) using the Bland Altman method for repeated measures [39] and mean differences between the two systems were estimated. Estimates of concurrent inter-method reliability were obtained by calculating intraclass correlation coefficients (ICC) of absolute agreement (ICC (2,1) A) and consistency (ICC (2,1) C) using a two-way random effects model [40]. To account for each individual being represented by measures from more than one repetition the ICC’s were estimated using a nested bootstrapping procedure [41]. In this procedure each resample was made from a reduced dataset where each subject was represented by one randomly selected trial. The number of resamples were set to 10,000 based on bootstrapping guidelines [42]. The reported ICC values are the averages of the 10,000 resamples. Confidence intervals for the ICC’s are based on the 2.5 and 97.5 percentiles of the bootstraps [42].

### Through range motion analysis

The analysis of concurrent validity of through range motion was performed by calculating the following: a repeated measures correlation (RMC) [43], LOA, root mean square errors (RMSE) between measurements, and mean differences between the two systems.

To minimize the influence of autocorrelation [39], we estimated LOA’s 100 times, with each estimate being based on a reduced dataset of five randomly selected observations. Each estimate was calculated by use of Bland and Altman’s procedure for repeated measures [39]. The reported LOA is the average LOA of the 100 estimates.

When calculating the RMSE, the multiple repeated measures from each participant were considered by using a mixed effect linear regression model. In this model, the Vicon measures were the dependent variable, the Captury measurements the independent variable and the identification numbers of the study participants were used as random effects. RMSE was calculated from the residuals of this model.

### Evaluation of agreement estimates

The Vicon system is accepted as state of the art equipment for assessing human movement, and the Plug-in Gait model is the most widely used and understood biomechanical model within the clinical and research community [44]. Nevertheless, the accuracy and precision of the system and the model is prone to limitations primarily caused by imprecise marker placements [2] and soft tissue artifacts (STA) [1, 45], and can as such not be considered a true gold standard. Consequently, the use of skin markers to describe knee joint motion must be presented with an envelope of accuracy, and standard errors of measurements of knee flexion in adults of 2.5° when walking and 6.3° when performing cutting maneuvers have been suggested [46].

Given our test procedure protocol with full range of motion and the uncertainty related to the translation of the Vicon Plug-in Gait model from adults to preschool children [28, 44], we find that a reasonable and conservative estimate of the effect of these errors on the precision of our Vicon measurements could be expressed as a SD of the error of 5°. Therefore, LOA between the two systems must be expected to have some width, and this creates a challenge in defining the cut-off points for accepted LOA.

To find these cut-off points, we used a novel pragmatic approach of simulating data of two systems (A and B) measuring the same construct in three scenarios with different SD’s of the error. In all three scenarios system A measured the construct with an error having a SD of 5°, while system B measured the construct with error SD’s of 5°, 7.5°, and 10°, depending on the scenario. Each scenario was conducted with 1000 trials, each containing 1000 observations by each of the two systems. Finally, we calculated LOA between system A and B for each scenario and averaged the LOA over the 1000 trials. The simulated average LOA estimates were ± 13.9°, ± 17.7°, and ± 21.9, and represent the LOA we could expect to find if the SD of the error of the Captury system was 5°, 7.5°, or 10° and if our assumption about the error of the Vicon system was correct. We then used these LOA estimates as cut-off values to interpret the LOA’s from our results and evaluating the Captury’s performance as being “good” (< ± 13.9°), “acceptable” (≥ ± 13.9° but < ± 17.7°), “questionable” (≥ ± 17.7° but < ± 21.9°), or “invalid” (≥ 21.9°). Because our main concern with this grouping was the level of precision, we based it on the span of the LOA (upper limit – lower limit), i.e. not taking the mean difference into account.

For the variables KHR, AHR and FPKD we had less literature to support the size of an a priori *error* assumption for the Vicon system. Hip joint center location is essential for these variables and has been found to be estimated with mean errors of 22 mm in normal children when using standard procedures [2]. Errors in the estimation of ankle joint centers and knee joint centers are less well described, but we assume these to be considerably smaller due to the easier identification of the bony landmarks used for reflective marker placement and the smaller amounts of soft tissue separating the reflective markers from the underlying bone. Based on this information, we assumed the SD of the errors for the Vicon for the KHR and AHR to be 0.2 and for FPKD to be 15 mm. By using the above simulation approach, LOA cut-points were found to be 0.55, 0.71, and 0.88 for KHR and AHR, and 42 mm, 53 mm, and 66 mm for FPKD.

### Evaluation of reliability estimates

Inter-method reliability expressed in terms of ICC estimates were evaluated as follows: values less than 0.5, between 0.5 and 0.75, between 0.75 and 0.9, and greater than 0.90 were interpreted as indicative of poor, moderate, good, and excellent, reliability respectively [47].

The analysis was done using R (R Core Team (2018)) [48], and the following R packages: tidyr [49], dplyr [50], stringr [51], purrr [52], ggplot2 [53], psych [54], lme4 [55], rmcorr [56], knitr [57], kableExtra [58], and here [59].

## Results

The participating children were between three and 6 years old, with a mean age of 4.8 years (SD 0.8), a mean height of 109.2 cm (SD 7.9), and a mean weight of 19.2 kg (SD 3.2). All children completed three trials of squats, but one squat trial from one of the children was due to technical issues not recorded by the Vicon system. The standing broad jump trials from one child were excluded since the child performed long steps with constant floor contact of at least 1 foot instead of jumping in all three trials. This leaves a data set of 41 trials of squats from 14 children and 39 trials of standing broad jumps from 13 children.

**Fig. 2:**
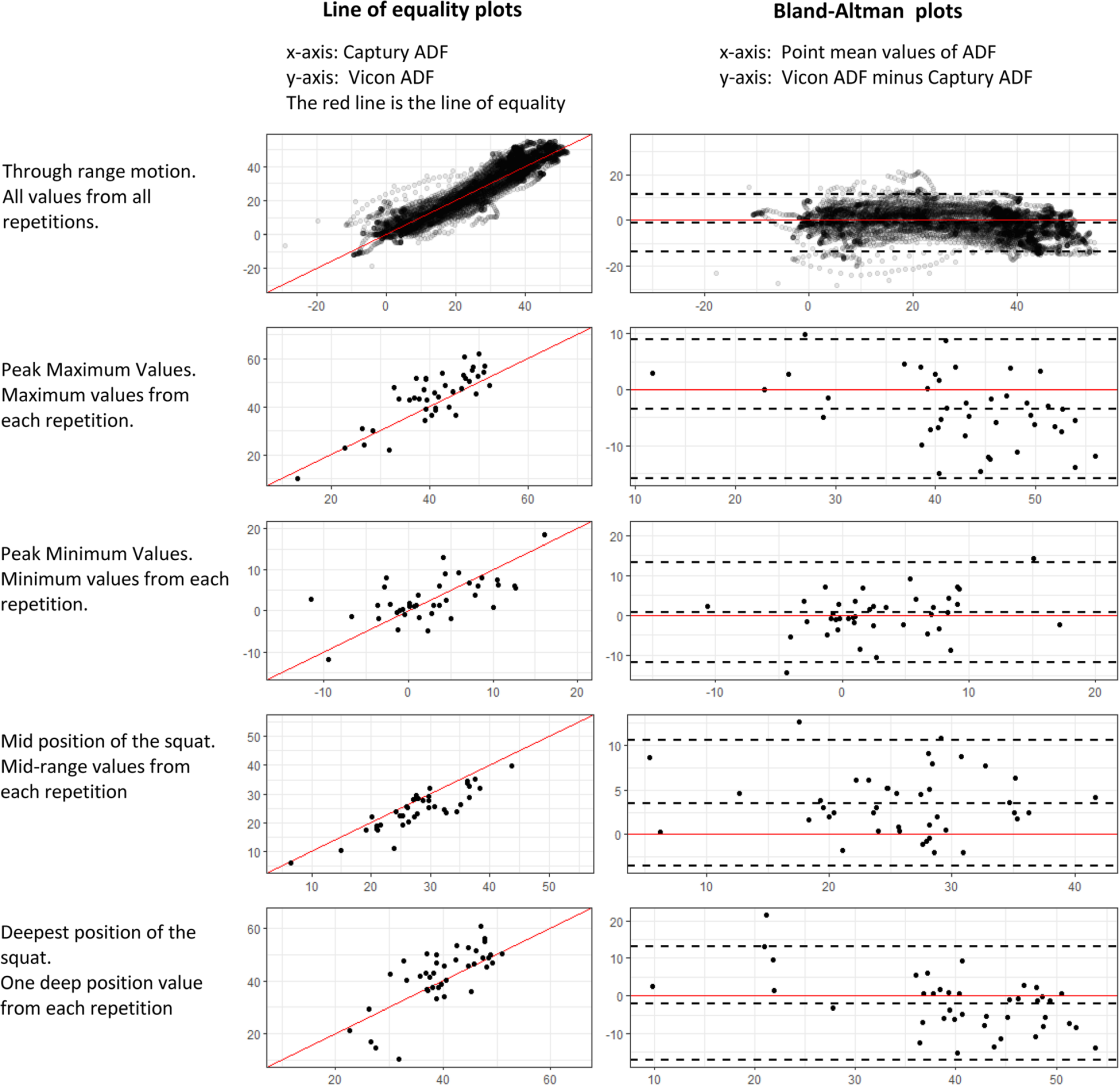
Agreement between the Captury and Vicon systems when measuring ankle dorsi flexion on 14 pre-school children performing three repetitions of squats. Note: ADF (Ankle Dorsi Flexion). Notes: Mid position of squat is defined as the frame during descent where knee-flexion is closest to being half of its peak value during the same repetition - I.e.*, the point where the child is halfway down.* The deepest position of squat is defined as the frame where knee flexion is equal to its peak values during that repetition

The visualization of the point, peak, and through range agreement between the two systems by use of a line of equality and Bland-Altman plots generally showed homoscedasticity, and no transformation of the data was performed. An example of these plots is supplied in Fig. 2.

**Fig. 3:**
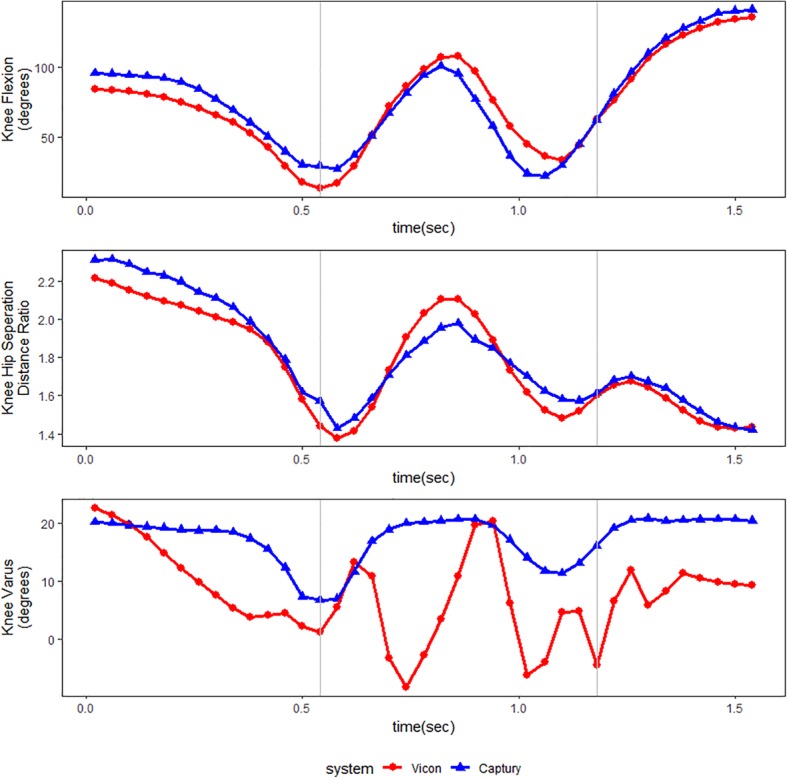
Measures of Knee Flexion, Knee Hip Separation Distance Ratio, and Knee Varus throughout a standing broad jump. The Grey vertical lines represent the moments of toe off and landing. A clear movement artifact is seen in the Vicon measures of Knee Varus. This artifact is believed to be caused by “wobbling” of the thigh wands in response to the rapid movements performed by the subject when setting off and landing

Due to knee valgus artifacts related to excessive wand movement of the plug-in gait marker set during take offs and landing the knee valgus measures for the standing broad jump trials were omitted*.* Figure 3 shows an example of one of these artifacts.

Descriptive statistics of jump height and jump length, along with the corresponding estimates of agreement are presented in Table 1. Correlation estimates were excellent ranging from 0.91 to 0.99, and LOA were found to be within − 6.6 and 5.2 cm.

**Table 1:** Descriptive statistics, agreement, and reliability of the Vicon and Captury systems when measuring jump height and length

		Descriptive Statistics				Agreement and Reliability				
Variable	System	min	mean	max	SD	LLoA	MD	ULoA	ICC(2,1) A	ICC(2,1) C
Jump Height	Vicon	−1.3^a^	7.4	16.4	4.0	−3.8	−0.8	2.2	0.91 [0.69;0.99]	0.93 [0.76;0.99]
	Captury	−1.8^a^	8.2	17.7	4.6					
Jump Length	Vicon	27.8	77.4	122.2	27.8	−6.6	−0.7	5.2	0.99 [0.98;1.00]	0.99 [0.99;1.00]
	Captury	26.0	78.4	124.6	27.3					

All values except intraclass correlations are in centimeters.

Note: *min* minimum, *max* maximum, *SD* standard deviation, *LLoA* lower limit of agreement, *MD* mean difference, *ULoA* upper limit of agreement, *ICC (2,1) A* intraclass correlation of absolute agreement, *ICC (2,1) C* intraclass correlation of consistency of agreement. 95% confidence intervals are presented in square brackets

^a^Jump height was calculated as the difference in height between the averaged position of the hip joints centers at the highest position of the jump and at toe off. This value will be negative if the subject has already begun descent at the first frame of the air phase

The results of through range concurrent validity are presented in Table 2. The RMC for Knee Varus and FPPA for the standing broad jumps were found to be low (RMC = 0.28 and 0.38), while the RMC values for the remaining variables indicated moderate to strong linear relationships (0.74 to 0.99 for squats, and 0.63 to 0.98 for standing broad jumps).

**Table 2:** Correlation and agreement estimates between the Captury and Vicon systems when measuring through range kinematics^a^ in squats and standing broad jumps

Variable	Range of Motion		Correlation and Agreement Estimates				
	Vicon	Captury	RMC	RMSE	LLoA	MD	ULoA
Squats							
Knee Flexion (°)	−21.7 to 146.0	−17.1 to 161.2	0.99	6.4	−25.1	−10.8	3.5
Hip Flexion (°)	−10.9 to 111.9	−19.6 to 125.2	0.92	11.4	− 32.9	−0.6	31.8
Ankle Dorsi Flexion (°)	−42.8 to 52.1	− 64.6 to 60.9	0.95	4.3	−13.5	− 1.0	11.5
Knee Varus (°)	−30.4 to 43.5	−28.8 to 20.8	0.28	5.9	−32.5	− 6.7	19.1
FP Projection Angle (°)	− 164.0 to 176.2	− 179.9 to 179.9	0.74	15.7	− 84.1	−5.9	72.3
FP Knee Angle (°)	−10.7 to 29.7	−16.6 to 37.5	0.83	3.7	−9.2	0.7	10.5
FP Knee Deviation (mm)	−34.2 to 146.0	−52.4 to 176.1	0.88	10.6	−33.6	−1.1	31.4
Knee-Hip SDR (ratio)	0.71 to 3.73	0.42 to 3.89	0.96	0.08	−0.36	0.01	0.38
Ankle-Hip SDR (ratio)	0.47 to 2.01	0.42 to 1.96	0.88	0.05	−0.26	0.03	0.32
Jumps							
Knee Flexion (°)	−17.0 to 136.9	−15.0 to 155.1	0.93	10.4	−26.4	−3.6	19.3
Hip Flexion (°)	−9.1 to 116.9	−16.6 to 141.0	0.77	16.9	−34.1	7.4	49.0
Ankle Dorsi Flexion (°)	−56.9 to 49.5	− 80.3 to 63.5	0.88	11.0	−32.4	3.6	39.6
FP Projection Angle (°)	−176.7 to 177.6	−179.2 to 178.6	0.38	20.3	−66.0	−0.2	66.4
FP Knee Angle (°)	−45.2 to 85.8	−47.7 to 90.7	0.75	6.6	−14.8	0.5	15.8
FP Knee Deviation (mm)	−33.1 to 81.9	− 67.8 to 149.7	0.63	12.6	−45.7	−1.0	43.8
Knee-Hip SDR (ratio)	0.67 to 3.29	0.42 to 3.71	0.95	0.11	−0.42	0.02	0.45
Ankle-Hip SDR (ratio)	0.53 to 5.83	0.57 to 6.55	0.98	0.13	−0.46	0.02	0.49

Note: Range of motion values reflects the minimum and maximum values observed across all trials

*FP* Frontal Plane, *SDR* Separation Distance Ratio, *RMC* Repeated Measures Correlation Coefficient, *RMSE* Root Mean Square Error, *LLoA* Lower Limit of Agreement, *MD* Mean Difference, *ULoA* Upper Limit of Agreement

^a^Through range kinematics specifies all points measured throughout the full range of motion

LOA’s from peak and point measures are presented in Table 3. In general, LOA’s were wide, and widest for standing broad jump measures. In comparison, most of the through range LOA’s reported in Table 2 are similar to the point and peak value LOA from the same variable. An exception to this trend is the wider LOAs for FPPA observed at more crouched positions (deepest position of squat [lower limit − 170.1°; upper limit 120.2°]; mid-position of squat [lower limit − 16.3°; upper limit 19.2°]; standing broad jump landing [lower limit − 47.9°; upper limit 45.5°]; standing broad jump deepest position [lower limit − 158.3°; upper limit 187.8°]).

**Table 3:** Concurrent reliability and agreement estimates between the Captury and Vicon systems when measuring lower extremity point and peak kinematics in squats and standing broad jumps

Compared Values	Agreement				Reliability	
	LLoA	MD	ULoA	Precision Span	ICC(2,1) A [CI]	ICC(2,1) C [CI]
Ankle Dorsi Flexion (°)						
Squats						
Maximum values	−16.0	−3.5	8.9	± 12.4^e^	0.69^c^ [0.23;0.93]	0.74^c^ [0.30;0.94]
Minimum values	−11.9	0.7	13.3	± 12.6^e^	0.62^c^ [0.06;0.92]	0.63^c^ [0.07;0.93]
Deepest position in squat	−17.0	−2.0	13.0	± 15.0^f^	0.68^c^ [0.26;0.92]	0.70^c^ [0.31;0.93]
Midpoint in descent	−3.5	3.5	10.5	± 7.0^e^	0.75^b^ [0.38;0.94]	0.85^b^ [0.55;0.97]
Jumps						
Maximum values	−22.6	−8.2	6.2	± 14.4^f^	0.38^d^ [0.00;0.69]	0.58^c^ [0.00;0.91]
Minimum values	−12.6	19.3	51.3	± 31.9^h^	0.22^d^ [0.00;0.51]	0.44^d^ [0.00;0.84]
Deepest position in landing	−22.7	−5.7	11.3	± 17.0^f^	0.62^c^ [0.00;0.96]	0.69^c^ [0.00;0.97]
Landing Impact	−15.1	5.5	26.1	± 20.6^g^	0.70^c^ [0.29;0.94]	0.76^b^ [0.39;0.96]
Ankle Hip Separation Distance Ratio (ratio)						
Squats						
Maximum values	−0.29	− 0.01	0.28	± 0.29^e^	0.89^b^ [0.72;0.97]	0.89^b^ [0.72;0.97]
Minimum values	−0.29	− 0.01	0.28	± 0.29^e^	0.86^b^ [0.59;0.98]	0.88^b^ [0.66;0.98]
Deepest position in squat	−0.27	0.01	0.30	± 0.29^e^	0.89^b^ [0.64;0.98]	0.89^b^ [0.66;0.98]
Midpoint in descent	−0.27	0.01	0.30	± 0.29^e^	0.91^a^ [0.78;0.98]	0.92^a^ [0.79;0.98]
Jumps						
Maximum values	−0.57	0.05	0.68	± 0.62^f^	0.94^a^ [0.82;0.99]	0.95^a^ [0.86;0.99]
Minimum values	−0.31	0.04	0.38	± 0.34^e^	0.73^c^ [0.00;0.96]	0.74^c^ [0.00;0.97]
Deepest position in landing	−0.41	0.07	0.55	± 0.48^e^	0.89^b^ [0.59;0.99]	0.90^a^ [0.63;0.99]
Landing Impact	−0.43	0.05	0.53	± 0.48^e^	0.95^a^ [0.74;0.99]	0.95^a^ [0.75;1.00]
Frontal Plane Knee Angle (°)						
Squats						
Maximum values	−11.0	−0.6	9.7	± 10.3^e^	0.88^b^ [0.73;0.95]	0.87^b^ [0.75;0.96]
Minimum values	−4.4	1.7	7.8	± 6.1^e^	0.65^c^ [0.11;0.94]	0.70^c^ [0.14;0.96]
Deepest position in squat	−11.0	3.3	17.6	± 14.3^f^	0.78^b^ [0.50;0.94]	0.82^b^ [0.56;0.95]
Midpoint in descent	−6.7	1.4	9.5	± 8.1^e^	0.78^b^ [0.34;0.95]	0.80^b^ [0.38;0.97]
Jumps						
Maximum values	−19.6	−1.2	17.3	± 18.5^g^	0.72^c^ [0.06;0.98]	0.73^c^ [0.06;0.99]
Minimum values	−7.2	2.1	11.4	± 9.3^e^	0.81^b^ [0.41;0.98]	0.84^b^ [0.47;0.99]
Deepest position in landing	−18.7	−0.3	18.1	± 18.4^g^	0.54^c^ [0.01;0.89]	0.56^c^ [0.01;0.90]
Landing Impact	−9.6	−0.1	9.4	± 9.5^e^	0.80^b^ [0.51;0.95]	0.81^b^ [0.53;0.95]
Frontal Plane Knee Deviation (mm)						
Squats						
Maximum values	−41.7	−8.6	24.6	± 33.2^e^	0.90^a^ [0.76;0.97]	0.92^a^ [0.79;0.98]
Minimum values	−18.1	4.3	26.7	± 22.4^e^	0.58^c^ [0.14;0.87]	0.61^c^ [0.15;0.88]
Deepest position in squat	−44.5	−0.8	42.9	± 43.7^f^	0.89^b^ [0.75;0.97]	0.90^b^ [0.76;0.97]
Midpoint in descent	−25.5	3.5	32.5	± 29.0^e^	0.85^b^ [0.59;0.96]	0.86^b^ [0.63;0.96]
Jumps						
Maximum values	−78.7	−13.7	51.4	± 65.1^g^	0.47^d^ [0.00;0.89]	0.51^c^ [0.00;0.91]
Minimum values	−18.9	9.5	38.0	± 28.4^e^	0.45^d^ [0.04;0.82]	0.53^c^ [0.06;0.87]
Deepest position in landing	−88.8	−3.4	82.0	± 85.4^h^	0.45^d^ [0.00;0.80]	0.46^d^ [0.00;0.81]
Landing Impact	−37.7	−1.6	34.4	± 36.0^e^	0.54^c^ [0.01;0.80]	0.56^c^ [0.01;0.82]
Frontal Plane Projection Angle (°)						
Squats						
Maximum values	− 142.2	−42.4	57.3	± 99.8^h^	0.52^c^ [0.06;0.91]	0.63^c^ [0.12;0.96]
Minimum values	−90.3	30.6	151.5	±120.9^h^	0.35^d^ [0.00;0.98]	0.38^d^ [0.00;0.98]
Deepest position in squat	−170.1	−24.9	120.2	±145.2^h^	0.57^c^ [0.00;0.91]	0.61^c^ [0.00;0.95]
Midpoint in descent	−16.3	1.5	19.2	± 17.8^g^	0.84^b^ [0.58;0.95]	0.85^b^ [0.62;0.98]
Jumps						
Maximum values	−84.3	−12.0	60.3	± 72.3^h^	0.62^c^ [0.00;0.97]	0.66^c^ [0.00;0.98]
Minimum values	−53.3	15.9	85.0	± 69.2^h^	0.44^d^ [0.03;0.99]	0.49^c^ [0.05;1.00]
Deepest position in landing	−158.3	14.8	187.8	±173.1^h^	0.37^d^ [0.00;0.94]	0.39^d^ [0.00;0.95]
Landing Impact	−47.9	−1.2	45.5	± 46.7^h^	0.51^c^ [0.00;0.83]	0.53^c^ [0.00;0.86]
Hip Flexion (°)						
Squats						
Maximum values	−34.4	2.5	39.5	± 37.0^h^	0.51^c^ [0.00;0.86]	0.53^c^ [0.00;0.86]
Minimum values	−34.4	−5.8	22.7	± 28.5^h^	0.47^d^ [0.00;0.81]	0.54^c^ [0.00;0.91]
Deepest position in squat	−32.1	3.9	40.0	± 36.0^h^	0.49^d^ [0.00;0.86]	0.51^c^ [0.00;0.87]
Midpoint in descent	−29.8	0.7	31.2	± 30.5^h^	0.60^c^ [0.15;0.85]	0.61^c^ [0.16;0.87]
Jumps						
Maximum values	−31.5	9.9	51.2	± 41.4^h^	0.25^d^ [0.00;0.62]	0.32^d^ [0.00;0.74]
Minimum values	−26.6	−2.7	21.2	± 23.9^h^	0.53^c^ [0.00;0.89]	0.55^c^ [0.00;0.90]
Deepest position in landing	−27.6	12.8	53.2	± 40.4^h^	0.50^d^ [0.08;0.80]	0.60^c^ [0.10;0.91]
Landing Impact	−21.5	14.8	51.1	± 36.3^h^	0.32^d^ [0.00;0.68]	0.45^d^ [0.00;0.86]
Knee Flexion (°)						
Squats						
Maximum values	−23.6	−11.7	0.2	± 11.9^e^	0.83^b^ [0.63;0.94]	0.96^a^ [0.89;0.99]
Minimum values	−27.9	−8.2	11.5	± 19.7^g^	0.52^c^ [0.10;0.79]	0.68^c^ [0.12;0.94]
Deepest position in squat	−23.6	−11.7	0.2	± 11.9^e^	0.83^b^ [0.63;0.94]	0.96^a^ [0.89;0.99]
Midpoint in descent	−11.5	−5.6	0.3	± 5.9^e^	0.84^b^ [0.64;0.94]	0.96^a^ [0.89;0.99]
Jumps						
Maximum values	−25.7	−5.6	14.6	± 20.1^g^	0.85^b^ [0.63;0.96]	0.89^b^ [0.68;0.97]
Minimum values	−23.2	−6.4	10.4	± 16.8^f^	0.55^c^ [0.15;0.81]	0.66^c^ [0.21;0.90]
Deepest position in landing	−23.6	−7.2	9.3	± 16.5^f^	0.91^a^ [0.76;0.98]	0.94^a^ [0.83;0.99]
Landing Impact	−25.8	−2.1	21.6	± 23.7^h^	0.74^c^ [0.15;0.96]	0.76^b^ [0.15;0.97]
Knee Hip Separation Distance Ratio (ratio)						
Squats						
Maximum values	−0.44	−0.03	0.38	± 0.41^e^	0.93^a^ [0.82;0.99]	0.93^a^ [0.82;0.99]
Minimum values	−0.18	0.08	0.34	± 0.26^e^	0.76^b^ [0.53;0.94]	0.82^b^ [0.62;0.96]
Deepest position in squat	−0.39	− 0.01	0.41	± 0.40^e^	0.94^a^ [0.86;0.99]	0.95^a^ [0.86;0.99]
Midpoint in descent	−0.39	−0.01	0.41	± 0.40^e^	0.88^b^ [0.63;0.98]	0.89^b^ [0.67;0.98]
Jumps						
Maximum values	−0.50	− 0.01	0.47	± 0.48^e^	0.87^b^ [0.63;0.97]	0.88^b^ [0.64;0.97]
Minimum values	−0.26	0.07	0.41	± 0.34^e^	0.69^c^ [0.29;0.91]	0.73^c^ [0.32;0.92]
Deepest position in landing	−0.39	0.05	0.48	± 0.44^e^	0.86^b^ [0.52;0.97]	0.87^b^ [0.54;0.98]
Landing Impact	−0.35	0.06	0.47	± 0.41^e^	0.89^b^ [0.61;0.99]	0.90^b^ [0.64;0.99]
Knee Varus (°)						
Squats						
Maximum values	−27.6	−6.5	14.7	± 21.1^g^	0.30^d^ [0.00;0.66]	0.38^d^ [0.00;0.73]
Minimum values	−19.5	−0.7	18.1	± 18.8^g^	0.45^d^ [0.00;0.78]	0.46^d^ [0.00;0.80]
Deepest position in squat	−37.3	−8.0	21.2	± 29.2^h^	0.29^d^ [0.00;0.72]	0.34^d^ [0.00;0.78]
Midpoint in descent	−37.4	−5.7	26.0	± 31.7^h^	0.33^d^ [0.00;0.70]	0.38^d^ [0.00;0.81]

*Note:*

*LLoA* Lower Limits of Agreement, *MD* Mean Difference, *ULoA* Upper Limits of Agreement, *ICC (2,1) A* Intraclass Correlation of Absolute Agreement, *ICC (2,1) C* Intraclass Correlation of Consistency of Agreement, *CI* 95% Confidence Interval.

Evaluation of intraclass correlation estimates: ^a^ Excellent; ^b^ Good; ^c^ Moderate; ^d^ Poor.

Evaluation of precision span estimates: ^e^ Good; ^f^ Acceptable; ^g^ Questionable; ^h^ Invalid.

**Fig. 4:**
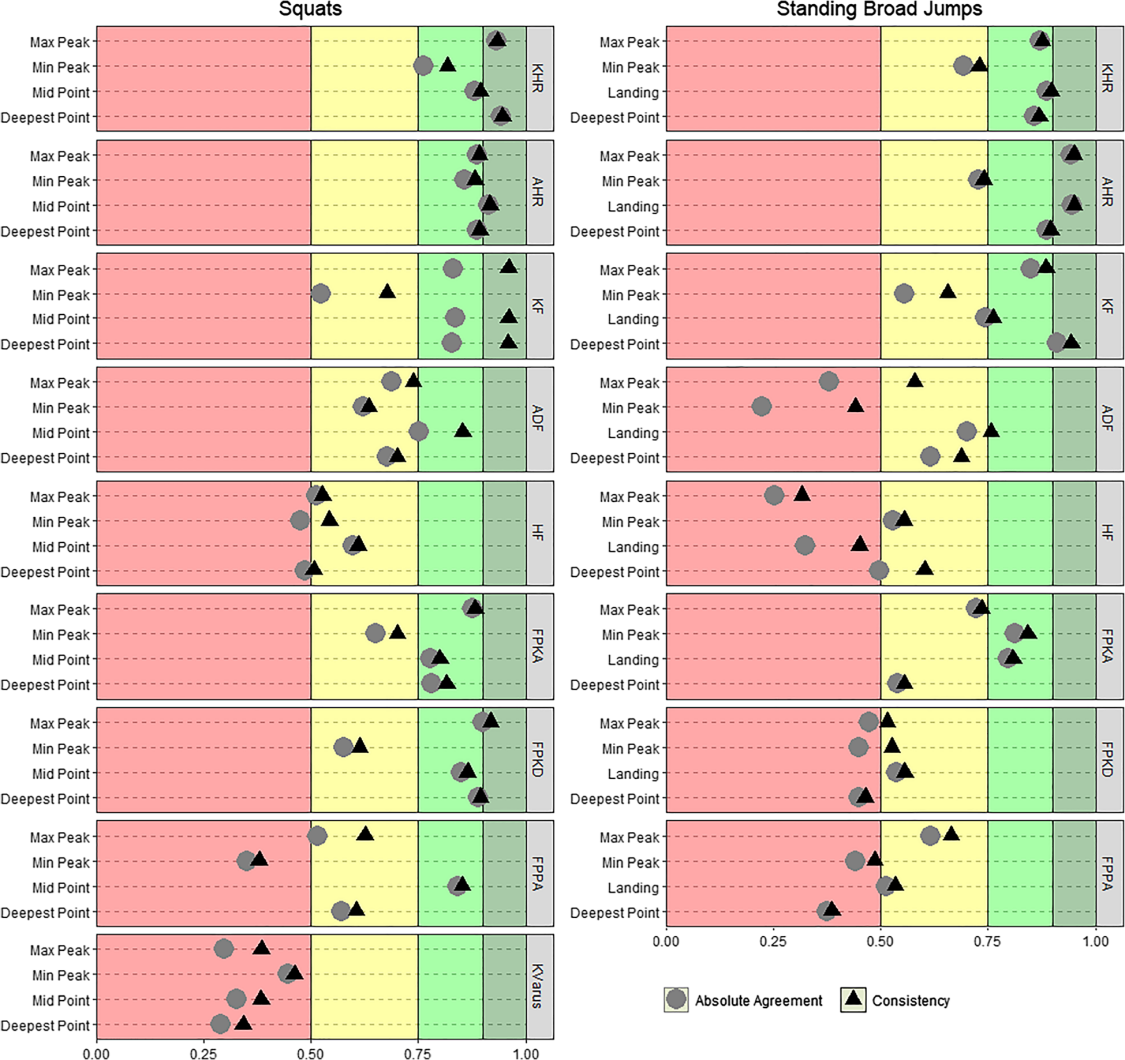
Intraclass correlation coefficients of absolute agreement and consistency between the Vicon and Captury systems. Note: KHR (Knee-Hip separation distance ratio), AHR (Ankle-Hip separation distance ratio), KF (Knee Flexion), ADF (Ankle Dorsi Flexion), HF (Hip Flexion), FPKA (Frontal Plane Knee Angle), FPKD (Frontal Plane Knee Deviation), FPPA (Frontal Plane Projection Angle), KVarus (Knee Varus). Range values used to group ICC values as either “poor” (< 0.5), “moderate” (0.5–0.75), “good” (0.75–0.9) or “excellent” (> 0.9) are marked by the background colors (red, yellow, light green, and dark green)

ICC estimates of absolute agreement and consistency for the kinematic variables are visualized in Fig. 4 and presented numerically in Table 3. ICC values ranged from 0.29 (Knee Varus measured at the deepest squat position) to 0.95 (AHR measured at the point of landing in standing broad jumps) and were, in general, higher for squats than standing broad jumps. For most of the variables, the differences between ICC (2,1) A and ICC (2,1) C were negligible, but a noticeable exception from this trend is knee flexion for squats were the consistency estimates were between 0.12 and 0.16 higher than the corresponding agreement estimates.

## Discussion

This study is, to our knowledge, the first to report on the concurrent validity of lower extremity kinematics and jump performance measures captured in preschool children using markerless motion capture technology. Our results suggest that this novel system can produce estimates of jump length, jump height, KHR, AHR, Knee flexion, FPKA, and FPKD with acceptable levels of agreement and reliability and thus warrants further investigations of their clinometric properties of measuring gross motor function in preschool children.

### Evaluation of agreement and reliability for jump height and length

The inter-method reliability for the performance measures, jump length and jump height, were found to be excellent, and LOA ranged from − 3.8 to 2.2 cm for jump height and − 6.6 to 5.2 cm for jump length (Table 3). We have not found other studies reporting on the validity of jump length and jump height measures in preschool children. While our LOA for jump length may seem wide, we believe our method is at least as accurate as manually measuring the children from a starting line, as our approach does not require the child to stand at a specific start position (i.e. a starting line), or to jump in a specific direction, and our approach thereby removes measurement error related to these issues.

### Evaluation of agreement and reliability for the kinematic variables

*The knee and ankle hip separation distance ratio* reliability estimates were excellent or good, except for minimum peak values that were found to be moderate. Furthermore, the accuracy was found to be acceptable with negligible mean differences [AHR between − 0.01 and 0.07; KHR between − 0.03 and 0.08] between the two systems, and precision estimates, based on our predefined cut-off values, were found to be good. We, therefore, consider estimates of AHR and KHR measured by the Captury system as valid.

*In knee flexion,* our results showed a substantial mean difference between the two systems [Between − 11.7° and − 2.1°] which was most pronounced for the squats where values of knee flexion measured by the Captury system were between 5.6° and 11.7° higher than the Vicon. Although not as extreme, similar results have been reported by Sandau et al. who also reported a markerless approach to measure higher values of knee flexion compared with marker-based data [11]. Moreover, marker-based systems have also been shown to underestimate knee flexion during stair ascent [60] and running [61] when compared to dynamic fluoroscopy. Contrary to the substantial mean differences, our study showed excellent to good inter-method reliability estimates for knee flexion, except for moderate minimum peak and landing values. Precision estimates ranged from good to invalid, with squat minimum peak values and jump maximum peak values being questionable, and values at the point of landing during jumping were found to be invalid. Visual inspection of the video footage suggests the minimum peak values were a result of the Captury system having difficulties with tracking fully extended knees as the hip joint centers were estimated too posteriorly. No feasible explanation to the differences at the point of landing was found.

*The ankle dorsi flexion* showed negligible mean differences for the squats [Between − 3.5° and 3.5°], precision estimates within the predefined cut-off for being either acceptable or good, and reliability estimates to be either moderate or good. For the standing broad jumps, mean differences were substantial [Between − 8.2° and 19.3°], and reliability estimates were mostly poor or moderate, with only the ICC (2,1) C landing estimate being good. Therefore, Ankle dorsi flexion measured by the Captury system cannot be considered valid per se, but selected time-points such as mid-point squat may be sufficiently valid to warrant further use.

*The hip flexion* mean differences were substantial [Between − 5.8° and 14.8°], precision estimates well beyond the predefined cut-off point for invalid, and reliability estimates were either poor or moderate. We suspect the poor estimates are a result of errors from the Captury system. Visual record examination showed that the hip joint center was placed too posteriorly in the standing position and that the differentiation of lumbar and pelvic motion was poor. We, therefore, consider hip flexion measures made by the Captury system as invalid.

*The frontal plane knee angle* mean differences between the two systems were negligible [Between − 1.2° and 3.3°]. Reliability estimates for FPKA were good or moderate. Precision estimates were generally good or acceptable, with only jump maximum peak estimates and deep landing estimates being questionable. Consequently, we consider estimates of FPKA measured by the Captury system as valid.

*The frontal plane knee deviation* showed that the mean differences were negligible [range: − 13.7 mm to 9.5 mm] and that precision estimates were mostly within our predefined limits of being good. Reliability estimates for the squats were mostly excellent or good, with only minimum peak values being moderate. Nevertheless, reliability estimates for the standing broad jumps were either moderate or poor, and it is, therefore, questionable to what extent FPKD deviation measures captured by the Captury system during jumping can be used in the future.

*For the frontal plane projection angle,* our results showed good squat mid-point reliability estimates. However, all other estimates of FPPA during the squats and standing broad jumps were either poor or moderate. Mean differences were substantial [Between − 42.4° and 30.6°], and precision estimates were with one exception well beyond our predefined cut-off point for being invalid and varied greatly between the different peak and point estimates. This was, in hindsight, unsurprising as the FPPA is highly affected by the height of the hip joint relative to the knee joint. At deep positions, the FPPA is exaggerated due to the low position of the hip joint, and the resultant frontal plane measurement error will, therefore, be magnified. Therefore, the FPPA is most likely only useful at low levels of knee flexion regardless of the system or method used to measure it.

*Knee varus* reliability estimates for the squats were poor, mean differences were mostly substantial [Between − 8.0° and − 0.7°], and precision estimates were mostly beyond our predefined cut-off point of being invalid. The Vicon varus measurements from the standing broad jumps were corrupted by movement artifacts (Fig. 3) and were therefore excluded, but we have no reason to believe that the Vicon varus measurements from the squats, used for the comparison, should have been corrupted as the movement was slower and without impacts. We, therefore, consider estimates of knee varus measured by the Captury as invalid.

### Comparison with other validations of markerless motion capture technology

Other reports on the accuracy and precision of kinematics captured by markerless motion capture systems have been made [11, 12, 62]. Results from these studies must be compared to ours with caution as they involve different age groups, are mostly concerned with gait analysis, use different marker-based biomechanical models and marker protocols, and different statistical approaches. Furthermore, most of these studies have been performed under conditions that are close to optimal for the markerless systems, as the studies, in general, make use of a controlled background setting [12], optimal light conditions [11, 62], and suits and/or caps for the subjects that improve the tracking quality [11, 12, 62].

The RMSE errors reported by Ceseracciu et al. for knee flexion (11.8°), hip flexion (17.6°) and ankle dorsi flexion (7.2°) [62] were somewhat wider than our findings for the through range squats, and comparable in size to our through range standing broad jump findings. Sandau et al. made a comparison study involving gait analysis performed on ten adults by a markerless system and a Vicon system with a more sophisticated biomechanical model than our plug-in-gait model [11]. Their findings (mean difference; SD of difference) for hip (− 0.4°; 2.6°), knee (2.8°, 3.5°) and ankle dorsi flexion (− 0.7°; 2.5°) are, both more precise and accurate than our findings. This may be explained by the above described differences in the experimental setup and their use of a more sophisticated biomechanical reference model, or by the fact that Sandau et al. transferred joint-center positions and segmental references frames from the marker-based to the markerless system in order to secure an identical [global] reference frame [11]. We did not transfer data between the two systems, and no effort was made to secure identical reference frames.

Outcomes from 2D measurement techniques have also been validated against 3D marker-based motion-capture systems [34, 63, 64]. Our method of generating 2D projections from 3D recordings are different from these approaches in that we project the positions of the knee, hip, and ankle joints onto the frontal plane of the recorded subject, whereas normal 2D approaches work with joint positions that are “projected” onto the view frustum of the camera recording the movements. Ortiz et al. compared 2D and 3D evaluations of knee valgus and reported concurrent measures of knee separation distance and knee-to-ankle separation ratio correlations of consistency of 0.94 and 0.96 [30] which are of similar size to the ones we have found for KHR and AHR.

### Strength and limitations

A strength of the study was the “field set-up” adapted with the Captury system, meaning that no special attention was made to optimize the background of the recording area, our recorded subjects did not wear special clothing to enhance the tracking quality, and the illuminance level of the recording area was quite low for recording purposes (246 lx). Therefore, our results do not reflect the optimal performance of the Captury system, but rather the performance one can expect outside of a laboratory environment where these parameters cannot be expected to be optimized, and our results are therefore generalizable to such settings.

This study contains several limitations discussed in the following.

The present validity is only provided for the analyzed functional tests (standing broad jumps and squats) and should not be generalized to other functional tests (including gait), populations, and age groups.

Practical and logistic issues limited the sample size to 14 children between three and 6 years of age. This is a small sample size, especially given that physical performance, motor skills, and morphology undergo large changes in this age-span, and this may impact the results.

The standard Plug-in-Gate model has been studied intensively in the literature, and the reliability has been determined on samples including children down to the age of 5 years [20, 21]. To our knowledge, the validity and reliability of the model have not been examined in ages below 5 years, and well-known issues with marker-based data, such as anatomical landmark recognition and STA, might be more pronounced in this age-group.

The global coordinate system of the Vicon and Captury were not aligned which, however, had no impact on the data since all selected outcome measures were based upon relative spatial positions. However, for future use it is recommended to align systems for easier interpretation of data, especially for absolute spatial data such as foot progression angle in gait analysis or if the computed jump length should be provided as a distance from a fixed point or line in the room.

Although the inter-system agreement and reliability estimates of jump length and jump height were excellent, we wish to note that the present comparisons of jump height and jump length do not provide absolute proof of validity, as the Vicon/Plug-in-Gate model is not a true gold standard. More work with comparing the motion-capture measures of jump length and jump height against more traditional and accepted methods is therefore needed before these motion-capture measures can be considered valid.

*A*lthough the Vicon system is considered *state-of-the-art* for non-invasive measurements of kinematics, the system is prone to substantial measurement error and its position as a true “gold standard” may be misleading. We have attempted to accommodate this by assuming an error SD of 5° degrees for all the kinematic variables measured by the Vicon system. Our interpretation of the precision results is highly affected by the size of this error assumption, and different assumptions would have led to different conclusions. It is not possible to measure the amount of the STA involved in our study, but STA is thought to be the prime cause for changes in distances between the hip joint center and knee joint center when using the Plug-in Gait model [44]. Ideally, the hip to knee distance should be constant, but under gait cycles, STA may cause this distance to change as much as 2 cm [44]. A post-hoc analysis to estimate the maximum change in the knee-joint-center to hip-joint-center distance throughout the squats revealed the mean of the maximum change to be 4.4 cm (SD 1.4 cm). This indicates that the STA’s that affected our data were at least comparable in size to that found in other studies, and our assumption of an error with a SD of 5° is therefore conservative.

### Future work

True gold-standards for assessing kinematics such as bone-anchored pins, percutaneous skeletal markers, or X-ray fluoroscopy are, due to their invasive nature, not available for use in children. Consequently, it is difficult to establish a true validation on the present technique, besides validating other functional tasks including gait. Standard clinimetric properties such as test-retest reliability, as well as the responsiveness of collecting data with the Captury system, needs to be established.

## Conclusion

The measurements by the markerless motion capture system “The Captury” cannot be considered interchangeable with the Vicon measures, but our results suggest that this novel system can produce estimates of jump length, jump height, KHR, AHR, Knee flexion, FPKA, and FPKD, with acceptable levels of agreement and reliability. These variables are promising for use in future research but require further investigation of their clinimetric properties.

## Data Availability

The dataset used and analyzed during the current study is available from the corresponding author on reasonable request.

## Abbreviations

2D: Two-Dimensional
3D: Three-Dimensional
AHR: Ankle to Hip separation distance Ratio
FPKA: Frontal Plane Knee Angle
FPKD: Frontal Plane Knee Deviation
FPPA: Frontal Plane Projection Angle
ICC (2,1) A: Intraclass Correlation of absolute agreement
ICC (2,1) C: Intraclass Correlation of consistency of agreement
ICC: Intraclass Correlation
KHR: Knee to Hip separation distance Ratio
LOA: Limits of Agreement
RMC: Repeated Measures Correlation
RMSE: Root Mean Square Error
SD: Standard Deviation
STA: Soft Tissue Artifact
